# Decline of Humoral Responses 6 Months after Vaccination with BNT162b2 (Pfizer–BioNTech) in Patients on Hemodialysis

**DOI:** 10.3390/vaccines10020327

**Published:** 2022-02-18

**Authors:** Michael Jahn, Johannes Korth, Oliver Dorsch, Olympia Evdoxia Anastasiou, Adalbert Krawczyk, Leonie Brochhagen, Lukas van de Sand, Burkhard Sorge-Hädicke, Bartosz Tyczynski, Oliver Witzke, Ulf Dittmer, Sebastian Dolff, Benjamin Wilde, Andreas Kribben

**Affiliations:** 1 Department of Nephrology, University Hospital Essen, University of Duisburg-Essen, 45147 Essen, Germany; johannes.korth@uk-essen.de (J.K.); bartosz.tyczynski@uk-essen.de (B.T.); benjamin.wilde@uk-essen.de (B.W.); andreas.kribben@uk-essen.de (A.K.); 2 KfH Kuratorium für Dialyse und Nierentransplantation e.V, Friesener Straße 37a, 96317 Kronach, Germany; oliver.dorsch@kfh.de; 3 Institute for Virology, University Hospital Essen, University of Duisburg-Essen, Virchowstr. 179, 45147 Essen, Germany; olympiaevdoxia.anastasiou@uk-essen.de (O.E.A.); ulf.dittmer@uk-essen.de (U.D.); 4 Department of Infectious Diseases, West German Centre of Infectious Diseases, University Hospital Essen, University of Duisburg-Essen, Hufelandstr. 55, 45147 Essen, Germany; adalbert.krawczyk@uk-essen.de (A.K.); leonie.brochhagen@uk-essen.de (L.B.); lukas.vandesand@uk-essen.de (L.v.d.S.); oliver.witzke@uk-essen.de (O.W.); sebastian.dolff@uk-essen.de (S.D.); 5 KfH Kuratorium für Dialyse und Nierentransplantation e.V, Alfried-Krupp-Str. 43, 45131 Essen, Germany; burkhard.sorge-haedicke@kfh.de

**Keywords:** BNT162b2, mRNA vaccines, anti-SARS-CoV-2 IgG, COVID-19, hemodialysis, neutralizing antibodies, chronic kidney disease

## Abstract

This study analyzed binding and neutralizing antibody titers up to 6 months after standard vaccination with BNT162b2 (two doses of 30 µg each) in SARS-CoV-2 naïve patients (n = 59) on hemodialysis. Humoral vaccine responses were measured before and 6, 12, and 24 weeks after the first vaccination. A chemiluminescent immunoassay (CLIA) was used to quantify SARS-CoV-2 IgG against the spike glycoprotein. SARS-CoV-2 neutralizing activity was tested against the wild-type virus. A multivariable binary regression model was used to identify risk factors for the absence of humoral immune responses at 6 months. At week 6, vaccine-specific seroconversion was detected in 96.6% of all patients with median anti-SARS-CoV-2 IgGs of 918 BAU/mL. At weeks 12 and 24, seroconversion rates decreased to 91.5% and 79.7%, and corresponding median binding antibody titers declined to 298 BAU/mL and 89 BAU/mL, respectively. Neutralizing antibodies showed a decay from 79.6% at week 6 to 32.8% at week 24. The risk factor with the strongest association for vanishing immune responses was low serum albumin (*p* = 0.018). Regarding vaccine-specific humoral responses 6 months after the standard BNT162b2 vaccination schedule, SARS-CoV-2 naïve patients receiving hemodialysis must be considered at risk of becoming infected with SARS-CoV-2 and being infectious.

## 1. Introduction

Patients with chronic kidney disease, in particular those being dependent on hemodialysis, are at high risk for fatal outcomes after infection with SARS-CoV-2 [1,2,3]. Additional risk factors for severe disease progression such as diabetes, higher age, cardiovascular diseases, or hypertension are common in these patients. Dialysis dependence is another important independent risk factor [2,4,5,6]. Moreover, it is recognized that these patients are particularly at risk for SARS-CoV-2 infection due to the difficulty of implementing hygienic concepts and quarantine restrictions in dialysis centers. Dialysis treatments cannot be discontinued and patients on hemodialysis are frequently attached to other health care institutions, such as nursing homes or outpatient services, and have higher hospitalization rates. Therefore, the scenario of a COVID-19 outbreak in a dialysis center always implies an involvement of other critical medical infrastructures and high mortality rates of severely ill patients [4,7]. The reported high morbidity and mortality rates led to a consequent implementation of preventive measures. These included the application measures to reduce the risk of infection (e.g., mandatory face masks, no food intake during dialysis, constant screening for COVID-19 symptoms, regular SARS-CoV-2 tests, isolation measures in cases of suspicious clinical symptoms) and the prioritization of patients on hemodialysis in vaccination programs, respectively [8].

Even though dialysis dependency is generally associated with hypo-responsiveness to conventional vaccination strategies [9,10,11,12], in April 2021, the first reports described high initial seroconversion rates after vaccination with the mRNA vaccine BNT162b2 [13,14]. Numerous other studies confirmed these humoral response rates after vaccination. Between 77% [15] to 98% [16] of mRNA-vaccinated patients on hemodialysis showed antibody responses estimated by binding antibody titers [5,15,16,17,18,19,20,21,22,23,24,25,26,27,28,29,30,31,32,33] or neutralizing assays [15,22,26,34]. This is a markedly higher response rate compared to immunocompromised patients with hematological malignancies or solid organ transplant recipients, who have shown initial response rates around 20–85% [35,36,37]. Nevertheless, when compared to healthy, non-dialysis controls, patients on hemodialysis displayed significantly lower antibody titers within the first 3 months after vaccination [5,13,14,15,17,21,22,23,24,25,26,28,33]. Given the higher age of dialysis patients (mean age of patients on hemodialysis in Germany is 68 years [38]), the hypo-responsiveness of these patients can be partly attributed to immune senescence [39]. Recently, however, Labriola and colleagues were able to show that dialysis dependence is an independent risk factor for reduced vaccine responses [5].

Facing the emergence of new coronavirus variants of concern (VOC), which have shown increased transmissibility and features of immune evasion such as reduced neutralization by vaccine-induced antibodies [40,41,42], knowledge about the actual immunoprotection and the decline of circulating vaccine-specific antibodies after a standard vaccination schedule is highly important for further decision-making processes concerning booster procedures. The aim of this study was to compare binding capacity and neutralization efficacy of vaccine-induced antibodies up to 6 months after vaccination with BNT162b2.

## 2. Materials and Methods

All patients on hemodialysis over 18 years of age, without immunosuppressive medication and without a reported prior SARS-CoV-2 infection, were recruited from the dialysis center in Kronach, Germany. Hygiene and screening measures to prevent COVID-19 outbreaks at this center included routine entry checks with measurement of body temperature and structured queries of COVID-19-associated symptoms at each dialysis, real-time PCR assays for SARS-CoV-2 RNA from nasopharyngeal swabs in the event of COVID-19 suspicion, and a complete PCR screening of all patients in May 2021.

Originally, 72 patients were enrolled for prospective analysis. In the further course of our evaluation, eight patients dropped out due to death, whereby only one of these cases was related to SARS-CoV-2-related disease (patient without seroconversion after two BNT162b2-vaccinations). One patient dropped out due to kidney transplantation. After screening for antibodies against the nucleocapsid protein at week 24, we excluded four patients who had signal-to-cutoff (S/CO) ratios over 0.8, which was considered indicative of previous occult or asymptomatic SARS-CoV-2 infections.

All subjects were vaccinated with the mRNA-based SARS-CoV-2 vaccine BNT162b2 (Pfizer–BioNTech) at a vaccination center in Kronach, Germany, according to the standard protocol (two doses of 30 µg administered 3–4 weeks apart) [43]. After informed consent from all participants was obtained, blood samples (7.5 mL S-Monovette^®^ Serum Gel, Sarstedt AG, Nümbrecht, Germany) for measurement of humoral vaccine responses were collected at the beginning of dialysis sessions at the following time points: before, as well as 6 weeks, 12 weeks, and 24 weeks after the first vaccination. The whole-blood samples were then centrifuged (medifuge 1215, Heraeus, Hanau, Germany) at 3500 rounds per minute for 30 min at a centrifugation temperature of 20 °C to collect serum. Patient characteristics, diagnoses, and laboratory values generally associated with low seroconversion rates after vaccination of patients with end-stage renal disease were recorded before the first vaccination. For this purpose, whole-blood samples (potassium EDTA for determination of leukocytes and hemoglobin, serum-gel for determination of C-reactive protein (CRP), vitamin D, serum albumin, parathormone, anti-HBs’ antibody titers; Sarstedt AG, Nümbrecht, Germany) were taken as part of the routine quality measurements of dialysis patients in Germany. For automated measurement of these laboratory parameters, the following devices were used: ARCHITECT Anti-HBs’ assay (Abbott Laboratories, Sligo, Ireland) for anti-Hbs-titers, ADVIA1 1800 Clinical Chemistry System (Siemens Healthcare Diagnostic, Erlangen, Germany) for CRP and serum albumin, ADVIA Centaur^®^ XPT Immunoassay-System (Siemens healthcare GmbH, Erlangen, Germany) for vitamin D and parathormone, and XN-1000 Pure (Sysmex, Norderstedt, Germany) for hemoglobin and leukocytes.

### 2.1. SARS-CoV-2 IgG Quantification Assays

For quantitative determination of the binding capacity of vaccination-induced anti-SARS-CoV-2 IgG, we used the chemiluminescent immunoassay (CLIA) LIAISON^®^ (Diasorin, Saluggia, Italy). This assay detects IgG antibodies against the anti-Trimeric Spike Glycoprotein of the SARS-CoV-2. According to the manufacturer’s recommendations, a binding antibody units’ (BAU/mL) ratio of <33.0 was considered to be negative and ≥33.8 to be positive. Note that 2080 BAU/mL is the upper limit of quantification of the CLIA. The CLIA was performed on all collected serum samples. Antibodies against the SARS-CoV-2 nucleocapsid protein were measured at week 24 with an enzyme-linked immunosorbent assay (ELISA) (Euroimmun Medizinische Labordiagnostika, Lübeck, Germany). According to the manufacturer’s recommendations, an S/CO ratio of <0.8 was considered negative, ≥0.8 to <1.1 borderline, and ≥1.1 positive. Only patients with S/CO < 0.8 were carried forward to further analysis. 

### 2.2. SARS-CoV-2 Neutralization Assay

The neutralizing capability of antibodies against SARS-CoV-2 was quantified using a previously described method [44,45]. The SARS-CoV-2 wild-type virus used in this study was isolated from a COVID-19 patient in April 2020 and included the D614G mutation. Serial dilutions (1:20–1:2560) of serum samples were incubated with 100 TCID_50_ of SARS-CoV-2 for 1 h at 37 °C and subsequently added to confluent Vero E6 cells cultured in 96-well microtiter plates. On day 2 after infection, cells were stained with crystal violet (Roth, Karlsruhe, Germany) and analyzed for the appearance of virus-induced cytopathic effects (CPE) by light microscopy. The neutralizing titer was defined as the reciprocal of the highest serum dilution at which no CPE breakthrough was observed in any of the triplicate cultures. The neutralization assays were conducted with the serum samples collected at 6 and 24 weeks after the first vaccination.

### 2.3. Statistical Analysis

Statistical analysis was performed using SPSS (version 21.0; SPSS Inc., Chicago, IL, USA) and GraphPad Prism (version 5.00; GraphPad Software Inc., San Diego, CA, USA). For descriptive statistics, absolute and relative frequencies were calculated for categorical parameters, whereas continuous parameters were characterized using the median (MD) as well as the first and third quartiles (Q1, Q3). Three groups were defined to compare humoral responses at 24 weeks after the initiation of the standard vaccination schedule with BNT162b2 as follows. Patients with anti-SARS-CoV-2 IgG < 33.8 BAU/mL and neutralizing antibody titer < 1:20 were classified as non-responders. Patients with anti-SARS-CoV-2 IgG ≥ 33 BAU/mL but neutralizing antibody titer efficacy < 1:20 were classified as insufficient responders. Patients with anti-SARS-CoV-2 IgG ≥ 33 BAU/mL and neutralizing antibody titer ≥ 1:20 were classified as responders. Inferential statistics to compare non-responders, insufficient responders, and responders included Fisher’s exact test for categorical variables and the Kruskal–Wallis test for continuous variables. Strength and direction of correlation between the quantified SARS-CoV-2 IgG antibody titers 24 weeks after the primary vaccination and different risk factors for low seroconversion rates were calculated using Spearman’s and Eta(n) correlation coefficients. The applied statistical tests were two-sided, and results were considered statistically significant when *p* < 0.05.

### 2.4. Analysis of Risk Factors

The risk factors of age, dialysis vintage, serum albumin as a surrogate parameter for malnutrition, CRP and leukocytes as surrogate parameters for inflammation, vitamin D, dialysis efficiency (Kt/V), body mass index (BMI), diabetes mellitus, parathormone, hemoglobin, and response to hepatitis B vaccination were selected according to previous studies on humoral vaccination responses of dialysis patients to hepatitis B, pneumococcus, or influenza vaccination [9,11]. Kt/V was calculated using the Daugirdas formula [46]. Hepatitis-B adequate vaccine response was defined at anti-HBs’ antibody titers of >10 U/L [47]. Diabetes mellitus was defined via antidiabetic medication (oral antidiabetics or insulin therapy). BMI was defined as dry weight in kilograms divided by height in square meters. Variables that correlated with *p* < 0.2 with anti-SARS-CoV-2 IgG level 24 weeks after the first vaccination were carried forward to binary logistic regression models to further analyze the association between these variables and negative vaccination responses in either the quantification or the neutralization assay. The risk factors were thereby analyzed separately from the SARS-CoV-2 IgG antibody levels at 6 and 12 weeks, since the measurement of antibody titers were not yet routinely recorded, in contrast to the hereby described patient characteristics. 

### 2.5. Ethics

The study was conducted according to the guidelines of the Declaration of Helsinki and approved by the ethics committee of the Medical Faculty of the University Duisburg-Essen (20-9753-BO).

## 3. Results

**Figure 1:**
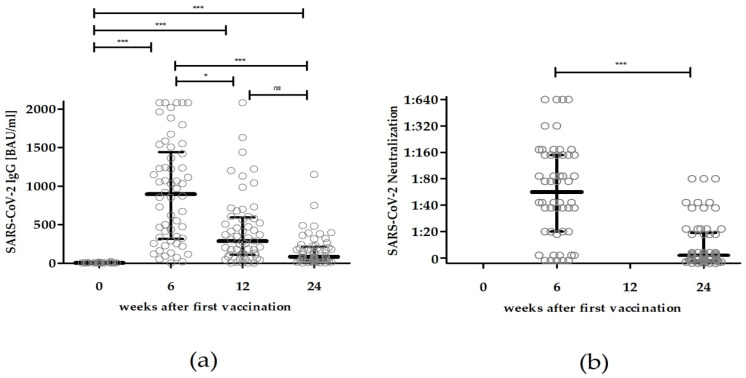
Dynamics of humoral immune responses in patients on hemodialysis after standard vaccination with two injections of 30 µg of BNT162b2. (**a**) Binding serum antibody titers determined after 0, 6, 12, and 24 weeks after the first vaccination. (**b**) Neutralizing antibody titers assessed after 6 and 24 weeks after the first vaccination. Circles represent the antibody titers of each subject; black bars represent median antibody titers with their corresponding interquartile ranges. Statistical analysis: Kruskal–Wallis Test followed by Dunn’s multiple comparison test; * = *p* < 0.05, *** = *p* < 0.001; ns = non-significant.

All 59 patients on hemodialysis who were evaluated had undetectable anti-SARS-CoV-2 IgG against the spike protein directly before the first vaccination as well as lacking anti-SARS-CoV-2 IgG against the nucleocapsid protein at week 24 (Figure 1 and Table 1). At the time of the first vaccination, the median [Q1; Q3] age of the finally included patients was 68 [59; 77] years, the median [Q1; Q3] dialysis vintage was 4 [2; 12] years, and the median [Q1; Q3] BMI was 27.1 [22.4; 30.3] kg/m^2^ (Table 2). The most common comorbidity was diabetes, which was diagnosed in 29 patients (49.2%). Response to prior Hepatitis B vaccination (titer of at least 1:10 U/L) was detectable in 16 patients (27.1%) at the time of enrollment (Table 2).

### 3.1. SARS-CoV-2 Binding Serum Antibody Titers after Vaccination with BNT162b2

Six weeks after the first dose of BNT162b2, 57 patients (96.6%) showed a vaccine-specific seroconversion with median anti-SARS-CoV-2 IgG [Q1; Q3] of 918 [322; 1505] BAU/mL. After 12 weeks, 54 patients (91.5%) showed measurable anti-SARS-CoV-2 IgG, with a median [Q1; Q3] of 298 [111; 605] BAU/mL (Figure 1, Table 1). Compared to the antibody titers 6 weeks after the first dose, five patients (8.0%) had a slight increment (maximum 1-fold increase in titer) and seven patients (11.3%) had an unchanged antibody titer. All others showed decreased antibody titers, most frequently a 2–2.9-fold decline (17/59 patients, 28.8%).

After 24 weeks, the number of patients with positive anti-SARS-CoV-2 IgG decreased to 47 (79.7%) and the median [Q1; Q3] dropped to 89 [38; 224] BAU/mL (Figure 1). Compared to the corresponding antibody titers 6 weeks after the first dose of BNT162b2, only one patient showed an increased and two patients showed an unchanged antibody titer. All other patients showed a significant decline of SARS-CoV-2 IgG levels. A total of 29 patients (49.2%) had a 7-fold or greater decrease in their titers compared to the antibody levels at week 6. All patients with anti-SARS-CoV-2 IgG > 918 BAU/mL at week 6 kept a positive antibody response in the quantification assay at week 24. 

The measured binding antibody titers at weeks 6 and 12 both correlated significantly with antibody levels at 24 weeks (Table 1). However, only the anti-SARS-CoV-2 IgG recorded at week 12 was significantly associated with lacking antibody responses after 24 weeks, both for non-detectable binding antibody titer (odds ratio 0.955 per 1 BAU/mL; 95% CI 0.913–0.998; *p* = 0.039) and for non-detectable neutralizing activity (odds ratio 0.996 per 1 BAU/mL; 95% CI 0.993–1.000; *p* = 0.037) (Table 3). Overall, a decrease of anti-SARS-CoV-2 IgG in this cohort was 67.5% between weeks 6 and 12 and 90.3% between weeks 6 and 24 after the initial vaccination with BNT162b2.

### 3.2. SARS-CoV-2 Neutralizing Antibody Titers after Vaccination with BNT162b2

**Figure 2:**
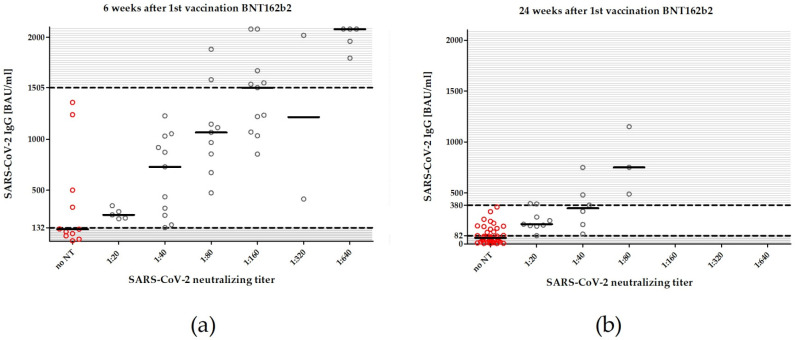
Association between binding and neutralizing antibodies at week 6 (**a**) and week 24 (**b**). Areas below the lower dashed lines indicate binding antibody titers without neutralizing activities (<132 BAU/mL at week 6 and <82 BAU/mL at week 24). Areas above the upper dashed lines indicate antibody titers definitely neutralizing the virus (≥1505 BAU/mL at week 6 and ≥380 BAU/mL at week 24). Areas between the dashed lines represent binding antibody levels for which no complete neutralizing activities can be assumed. At week 6, 87.8% of all patients with binding antibody levels between 132 to 1360 BAU/mL showed neutralizing activities against SARS-CoV-2. At week 24, 43.5% of all patients with binding antibody levels between 82 to 362 BAU/mL neutralized the virus. Red circles indicate no neutralizing activity, gray circles indicate neutralizing activity.

Six weeks after the first dose of BNT162b2, 43 of 54 patients (79.6%) harbored neutralizing antibodies against SARS-CoV-2 with a median [Q1; Q3] neutralization titer of 1:80 [1:20; 1:160]. The quantified anti-SARS-CoV-2 IgG and the neutralizing titer at week 6 were related as follows (Figure 2). Neutralizing activity was not measurable in any patient with anti-SARS-CoV-2 IgG < 132 BAU/mL (0 of 7 patients). In patients with anti-SARS-CoV2 IgG between 132 to 1360 BAU/mL, neutralizing antibodies were detectable in 29 of 33 patients (87.8%). In all patients with anti-SARS-CoV-2 IgG ≥1505 BAU/mL, neutralizing antibodies were detectable (14 of 14 patients). 

Twenty-four weeks after initial vaccinations with BNT162b2, neutralizing antibodies were detected in only 19 of 58 patients (32.8%) and the median [Q1; Q3] neutralization titer for the overall patient cohort decreased to 0 [0; 1:20]. The anti-SARS-CoV-2 IgG and the neutralization titers showed the following associations at week 24 (Figure 2). None of the patients with anti-SARS-CoV-2 IgG < 82 BAU/mL had neutralizing antibodies (0 of 27 patients). For patients with anti-SARS-CoV-2 IgG between 82 and 362 BAU/mL, neutralizing antibodies were detectable in 43.5% (10 of 23 patients). All patients with antibody titers ≥ 380 BAU/mL harbored neutralizing antibodies (8 of 8 patients).

### 3.3. Risk Factors for Antibody Titers <33.8 BAU/mL 24 Weeks after the First Vaccination

Based on clinical data and laboratory values at the time of the first vaccination, age (−0–411; *p* < 0.001), diabetes mellitus (−0.256; *p* = 0.053), and CRP (−0.319; *p* = 0.015) correlated inversely, and vitamin D (0.361; *p* = 0.006), positive response to hepatitis B vaccination (0.268; *p* = 0.042), hemoglobin (0.219; *p* = 0.098), and serum albumin (0.466; *p* < 0.001) correlated correspondingly with the detection of anti-SARS-CoV-2 IgG at week 24 (Table 2). However, in a regression model, serum albumin showed the strongest association with negative anti-SARS-CoV-2 IgG at week 24 (odds ratio 0.0.965 per 0.1 g/L; 95% CI 0.937 to 0.994; *p* = 0.017), while none of these factors was significantly associated to negative results in the neutralization assay (Table 4).

## 4. Discussion

The present study demonstrates that the measurable humoral responses after vaccination with BNT162b2 decreased consecutively over time in patients on hemodialysis. Six months after the initial vaccination with BNT162b2, humoral protection was diminished significantly in the majority of patients on hemodialysis with only 32.8% of patients having neutralizing antibodies. Furthermore, anti-SARS-CoV-2 IgG levels declined sharply with negative binding antibody responses in 20.3% of all patients. Thus, it must be assumed that 6 months after first vaccination, patients on dialysis are at risk for SARS-CoV-2 infection and, thus, being infectious. 

To date, long-term data on SARS-CoV2-vaccinated patients are scarce, especially in the vulnerable group of patients on hemodialysis. A progressive decline over time of circulating SARS-CoV-2-specific antibodies has been described in several other patient cohorts. Convalescent patients on hemodialysis have shown significant decays of SARS-CoV-2-specific antibodies, but still 85% of these patients kept seropositivity 6 months after infection [48,49]. Likewise, non-dialysis-dependent subjects have shown declining but persistent humoral responses of binding and functional antibodies against different SARS-CoV-2 variants over 6 months after mRNA vaccinations against COVID-19 [50,51,52,53,54]. Risk factors that appear to contribute to greater decline in antibody titers 6 months post-vaccination are older age [55,56] and a combination of more than two chronic conditions such as diabetes, smoking, obesity, heart disease, or chronic lung diseases [56], which are common comorbidities in patients on hemodialysis.

However, waning of antibody levels in patients on hemodialysis after a standard vaccination schedule with two doses of BNT162b2 appears particularly fast as indicated by our study data. The recently reported data by Davidovic et al. [57] can be confirmed by our findings. The authors used the same CLIA (LIAISON^®^, Diasorin, Saluggia, Italy) and measured a decrease of the median anti-SARS-CoV-2 IgG from 1110 BAU/mL at week 4 to 85.6 BAU/mL at week 24 after the second vaccination with BNT162b2. Their rate of patients with detectable virus neutralization reached 50.6% at 6 months after application of the second dose of BNT162b2. 

Our study is a first follow-up of an mRNA-vaccinated dialysis cohort over 6 months describing vaccine-specific binding and neutralizing antibody responses at different time points. By excluding factors such as immunosuppressive medication, heterologous vaccination, or prior SARS-CoV-2 infections, which all are known to have a great influence on immune responses after vaccination [16,17,22,24,26,27,28,30,36], this study focused particularly on the interaction between vaccination and hemodialysis dependence. The recruitment of this homogenous patient cohort comes at the expense of the smaller sample size. While previous exposure to SARS-CoV-2 significantly increases the immunogenicity of subsequent vaccinations and contributes to a long-term persistency of humoral responses [5,18,19], patients on immunosuppressants after kidney transplantation have shown low vaccination response rates over 4 months post-vaccination [58,59]. To the best of our knowledge, this is the first study that excluded occult and inapparent SARS-CoV-2 infections in patients on hemodialysis at several levels and over time. It is a strength of our study that we described a SARS-CoV-2 naïve cohort that had not been infected with SARS-CoV-2 and whose humoral responses can be interpreted as vaccine induced.

One key aspect of our study is the functional assessment of antibodies in a neutralization assay. In this assay, neutralization activity against the wild-type strain was tested and the majority of patients lost neutralization capacity over time. Against different variants of concern (VOC), such as the currently prevalent B.1.617.2 (also termed Delta variant), vaccine-generated antibodies have shown lower efficacy [34,40,60], which is also expected for the variant B.1.1.529 (Omicron variant) [61]. Thus, it must be assumed that, in the current pandemic dynamics, our neutralizing assay rather overestimates the actual vaccine-induced humoral immune protection in our cohort.

The success of vaccination is of utmost importance for the control of SARS-CoV-2 infections in dialysis centers. As it became increasingly recognized that vaccine-induced neutralizing antibodies can be associated with protection against initial infections [62], vaccination became a prioritized measure to create immunity against SARS-CoV-2 infection in the complex care setting of dialysis centers. However, the well-known hypo-responsiveness of patients on hemodialysis to other vaccines, such as hepatitis B, pneumococcus, or influenza [9,10,11,12], led to skepticism about the actual efficacy of COVID-19 vaccines in dialysis settings in general and for the use of novel mRNA vaccines in particular.

A variety of factors have been discussed as being responsible for the anergic immune reactions of patients receiving dialysis, leading to impaired responses of the innate (neutrophil and monocyte function) and adaptive (B- and T-cell-mediated responses, antigen processing) immune system [63,64,65,66]. Uremic toxins interfere with the immune system by, for example, altering physiological processes of hormones, enzymes, antibodies, lipoproteins, or transport proteins, which leads to complexly disturbed metabolic activities [66]. Low-grade inflammatory processes are permanently activated due to reduced renal elimination of pro-inflammatory cytokines as well as recurrent infections, increased oxidative stress, volume overload, bioincompatibility of dialysis products, or dialysate-associated endotoxin exposures [63,66,67]. Both inflammation and uremia are not only associated with perturbed immune responses but also to malnutrition and protein-energy wasting in patients on hemodialysis [63,66,68]. Accordingly, serum albumin as a surrogate parameter for malnutrition has been repeatedly identified as a risk factor for poor seroresponses after vaccination against SARS-CoV-2-related diseases [27,29,33]. Similarly, we identified serum albumin as the most substantial factor in determining negative anti-SARS-CoV-2 IgG 6 months later with a risk increase of 3.5% per 0.1 g/L drop. However, inflammatory processes were only described on the basis of CRP and leukocytes. There may be more suitable parameters for this purpose (e.g., IL-6, IL-1, TNF alpha).

Under consideration of waning antibody levels, we must assume that patients on hemodialysis can be infected and be infectious again the longer it has been since their last vaccination. Therefore, non-pharmacological measures, such as physical distancing, regular testing, generous spatial isolation in suspicious cases, or wearing of facial masks remain mainstays of preventing SARS-CoV-2-related diseases in routine clinical practice [69].

As recommended for other vaccination schedules [70], a modification of the standard strategy must also be taken into account to prolong the mRNA vaccine’s protective antibody effects in patients on hemodialysis. Vaccination strategies including three doses of BNT162b2 were carried out in France. It was shown that, compared to the median anti-S IgG antibodies after the second dose, a third dose enhanced humoral responses again by a 6- to 10-fold increase. Furthermore, in almost half of initial non-responders, the application of a third dose of BNT162b2 elicited detectable humoral responses [19,20,32,71]. A dose–response relationship, which shows that the more vaccine is inoculated, the higher the immunogenicity, can also be reasoned from substance comparisons between BNT162b2 and mRNA-127. In addition to aspects such as better thermostability, lipid formulations, or mRNA modifications [72,73], above all the three times higher dose of mRNA-172 (100 µg vs. 30 µg of mRNA content) might explain the higher immunogenicity observed in patients on hemodialysis vaccinated with the standard vaccination schedules [27,28].

The long-term persistence of humoral protection and actual efficacy of such modified vaccination strategies still needs to be investigated. However, such data imply that booster immunizations can counteract the waning of vaccine-specific immunity over time in patients receiving dialysis.

To date, decision-making processes regarding COVID-19 vaccination schedules and the estimation of vaccine responsiveness are primarily driven by assessments of antibodies. In healthy subjects, the decline of antibody titers seems faster than the actual decline of vaccine efficacy [43,52,74], implicating further immune functions beyond the humoral responses as being relevant for vaccine-induced immunoprotection. It has already been suggested that activated T-cells still could limit disease progressions even when neutralizing activities are low [75,76]. Recently, the determination of spike-specific CD4+ T cells and spike-specific T follicular helper cells were connected to viral neutralizing capacities, showing that these cells are crucial to differentiate B cells into antibody-producing plasma cells [71]. Furthermore, the profiling of spike-specific memory B-cells was suggested to more precisely indicate the capacity of humoral responses in the case of a pathogen encounter [50]. The evaluation of such complex immune responses could help to better predict the vaccine-induced immunogenicity more comprehensively and better classify antibody thresholds that will protect against severe SARS-CoV-2-related diseases.

## 5. Conclusions

The measurable humoral response after vaccination with BNT162b2 with a two-shot standard vaccination schedule decreased consecutively over time in patients on dialysis. Six months after initial vaccination, only 32.8% of patients showed persistence of neutralizing antibodies combined with a sharp decline of anti-SARS-CoV-2 IgG levels as measured by CLIA. Thus, SARS-CoV-2 naïve patients receiving hemodialysis are at risk for infection with SARS-CoV-2 and, thus, being infectious if a vaccination scheme with two doses of 30 µg of BNT162b2 is applied. 

## Figures and Tables

**Table 1:** Quantified antibodies of patients on hemodialysis throughout 24 weeks after the first vaccination.

Variable	All	Non-Responder	Insufficient Responder	Responder	Subgroup Comparison	Correlation with Anti -SARS-CoV-2 IgG 24 Weeks after 1st Vac
	MD [Q1;Q3], (Range: Min–Max) or n (% of Subgroup)					
Patients	59	12	28	19	NA	NA
SARS-CoV-2 IgG against the nucleocapsid protein, *S/CO ratio*						
24 weeks after 1st vac	0.05 [0.03; 0.10],	0.07 [0.04; 0.11],	0.05 [0.04; 0.15],	0.04 [0.04; 0.06],	*p* = 0.463 *	−0.175; *p* = 0.194 ^†^
	(0.02–0.47)	(0.02–0.17)	(0.02–0.47)	(0.03–0.23)		
SARS-CoV-2 IgG against the spike protein, *BAU/mL*						
before 1st vac	5 [5; 5],	5 [5; 5],	5 [5; 5],	5 [5; 5],	*p* = 0.844 *	0.089; *p* = 0.506 ^†^
	(5–22)	(5–6)	(5–12.5)	(5–22)		
6 weeks after 1st vac	918 [322; 1505],	172 [58; 586],	823 [364; 1127],	1794 [1222; 2080],	*p* < 0.001 *	0.669; *p* < 0.001 ^†^
	(5–2080)	(5–871)	(132–1672)	(117–2080)		
12 weeks after 1st vac	298 [111; 605],	44 [5; 85],	265 [175; 414],	723 [497; 1275],	*p* < 0.001 *	0.918; *p* < 0.001 ^†^
	(5–2080)	(5–132)	(54–1040)	(130–2080)		
24 weeks after 1st vac	89 [38; 224],	13 [5; 23],	78 [57; 172],	292 [183; 482],	*p* < 0.001 *	NA
	(5–1150)	(5–30)	(34–362)	(82–1150)		

Non-responder = anti-SARS-CoV-2 IgG < 33.8 BAU/mL and neutralizing antibody titer < 1:20; insufficient responder = anti-SARS-CoV-2 IgG ≥ 33.8 BAU/mL but neutralizing antibody titer efficacy < 1:20; responder = anti-SARS-CoV-2 IgG ≥ 33.8 BAU/mL and neutralizing antibody titer ≥ 1:20; vac = vaccination; MD = median; Q1 = 1st quartile; Q3 = 3rd quartile; n = count; NA = not applicable; * = Kruskal–Wallis test. ^†^ = Spearman’s correlation coefficient.

**Table 2:** Characteristics of patients on hemodialysis at time point of first vaccination and their further serological responses throughout the following 24 weeks.

Variable	All	Non-Responder	Insufficient Responder	Responder	Subgroup Comparison	Correlation with Anti -SARS-CoV-2 IgG 24 Weeks after 1st Vac
	MD [Q1;Q3], (Range: Min–Max) or n (% of Subgroup)					
Patients	59	12	28	19	NA	NA
Gender, n (%)	♀ 22 (37.3%)	♀ 6 (50.0%)	♀ 7 (25.0%)	♀ 9 (47.4%)	*p* = 0.156 °	0.112, *p* = 0.401 ^‡^
	♂ 37 (62.7%)	♂ 6 (50.0%)	♂ 21 (75.0%)	♂ 10 (52.6%)		
Age, years	68 [59; 77],	76 [66; 78],	68 [57; 76],	62 [54; 70],	*p* = 0.034 *	−0.411; *p* < 0.001 ^†^
	(50–90)	(63–85)	(53–83)	(50–90)		
Body Mass Index, kg/m²	27.1 [22.4; 30.3],	27.6 [23.5; 30.3],	26.8 [21.5; 29.9],	27.0 [23.5; 31.6],	*p* = 0.819 *	0.037; *p* = 0.786 ^†^
	(17.9–46.3)	(19.0–36.7)	(18.4–42.8)	(17.9–46.3)		
Dialysis vintage, years	4 [2; 12],	4 [1; 10],	4 [2; 9],	4 [1; 26],	*p* = 0.892 *	0.118; *p* = 0.376 ^†^
	(0–46)	(0–46)	(0–31)	(0–38)		
HepB- vac responders	16 (27.1 %)	2 (16.7 %)	6 (21.4 %)	8 (42.1 %)	*p* = 0.239 °	0.268; *p* = 0.042 ^†^
Diabetes mellitus	29 (49.2 %)	7 (58.3 %)	13 (46.4 %)	9 (47.4 %)	*p* = 0.838 °	−0.256; *p* = 0.053 ^†^
C-reactive Protein, mg/dL	3.4 [1.8; 9.4],	4.9 [2.4; 22.6],	4.5 [2.0; 10.7],	2.3 [0.9; 4.5],	*p* = 0.036 *	−0.319; *p* = 0.015 ^†^
	(<0.4–60.7)	(2.2–22.6)	(0.6–60.7)	(<0.4–11.2)		
Leukocytes, e^3^/µL	6.7 [5.6; 8.0],	6.8 [5.7; 9.4],	6.8 [5.4; 8.1],	6.6 [5.8; 7.8],	*p* = 0.729 *	−0.081; *p* = 0.544 ^†^
	(1.6–11.7)	(5.3–11.7)	(1.6–11.0)	(4.3–10.7)		
Vitamin D, ng/mL	23.4 [16.2; 32.0],	15.2 [11.0; 22.6],	24.3 [16.5; 30.5],	27.0 [20.8; 35.0],	*p* = 0.015 *	0.361; *p* = 0.006 ^†^
	(9.4–45.7)	(9.4–37.9)	(9.6–45.7)	(15.4–45.3)		
Serum albumin, g/dL	3.9 [3.8; 4.2],	3.7 [3.1; 3.9],	3.9 [3.8; 4.2]	4.2 [3.9; 4.3],	*p* = 0.007 *	0.466; *p* < 0.001 ^†^
	(2.6–4.5)	(2.6–4.3)	(3.5–4.4)	(3.6–4.5)		
Kt/V	1.47 [1.22; 1.70],	1.45 [1.13; 1.67],	1.45 [1.19; 1.66],	1.58 [1.24; 1.92],	*p* = 0.564 *	0.168; *p* = 0.207 ^†^
	(0.91–2.19)	(0.99–1.81)	(0.91–2.05)	(0.94–2.19)		
Parathormone, pmol/L	19.3 [11.5; 30.3],	22.5 [14.2; 32.2],	18.8 [14.0; 30.2],	14.8 [8.2; 27.3],	*p* = 0.706 *	−0.121; *p* = 0.367 ^†^
	(1.1–59.7)	(3.4–42.6)	(4.7–46.2)	(1.1–59.7)		
Hemoglobin, g/dL	11.5 [10.8; 12.6],	10.9 [10.4; 11.9],	11.3 [11.0; 12.2],	12.1 [11.2; 12.7],	*p* = 0.104 *	0.219; *p* = 0.098 ^†^
	(9.2–13.9)	(10.0–13.9)	(9.2–13.7)	(10.5–13.3)		

Non-responder = anti-SARS-CoV-2 IgG < 33.8 BAU/mL and neutralizing antibody titer < 1:20; insufficient responder = anti-SARS-CoV-2 IgG ≥ 33.8 BAU/mL but neutralizing antibody titer efficacy < 1:20; responder = anti-SARS-CoV-2 IgG ≥ 33.8 BAU/mL and neutralizing antibody titer ≥ 1:20; vac = vaccination; MD = median; Q1 = 1st quartile; Q3 = 3rd quartile; n = count; NA = not applicable; * = Kruskal–Wallis test; ° = Fisher’s exact test; ^‡^ = Eta(n) correlation coefficient; ^†^ = Spearman’s correlation coefficient.

**Table 3:** SARS-CoV-2 IgG 6 and 12 weeks after the first vaccination and their associated risk for negative humoral responses.

Variable	Absence of SARS-CoV-2 IgG 24 Weeks after 1st Vac	Absence of Neutralizing Antibodies 24 Weeks after 1st Vac
	Adjusted Odds Ratio (95% CI); Significance	Adjusted Odds Ratio (95% CI); Significance
SARS-CoV-2 IgG, 6 weeks after 1st vac per 1 BAU/mL	1.002 (0.996–1.008); *p* = 0.540	0.998 (0.997–1.000); *p* = 0.120
SARS-CoV-2 IgG, 12 weeks after 1st vac per 1 BAU/mL	0.955 (0.913–0.998); *p* = 0.039	0.996 (0.993–1.000); *p* = 0.037

SARS-CoV-2 IgG = Severe Acute Respiratory Syndrome-Corona Virus type-2 Immunoglobulin G; vac = vaccination.

**Table 4:** Clinical and laboratory factors and their associated risk for negative humoral responses.

Variable	No SARS-CoV-2 IgG 24 Weeks after 1st Vac	No Neutralizing Antibodies 24 Weeks after 1st Vac
	Adjusted Odds Ratio (95% CI); Significance	Adjusted Odds Ratio (95% CI); Significance
Age per 1 year	1.056 (0.962–1.160); *p* = 0.249	1.026 (0.960–1.096); *p* = 0.450
CRP per 1 mg/dL	1.017 (0.952–1.087); *p* = 0.617	1.127 (0.948–1.341); *p* = 0.176
Serum albumin per 0,1 g/dL	0.965 (0.937–0.994); *p* = 0.017	0.988 (0.963–1.014); *p* = 0.356
Vitamin D per 1 ng/mL	0.912 (0.816–1.019); *p* = 0.105	0.963 (0.893–1.039); *p* = 0.334
Positive hepatitis B vac response	0.514 (0.061–4.338); *p* = 0.541	0.263 (0.057–1.201); *p* = 0.085
Hemoglobin per 1 g/L	1.217 (0.513–2.888); *p* = 0.656	0.732 (0.364–1.471); *p* = 0.381
Diabetes mellitus	1.517 (0.280–8.207); *p* = 0.629	0.467 (0.107–2.034); *p* = 0.311

SARS-CoV-2 IgG = Severe Acute Respiratory Syndrome-Corona Virus type-2 Immunoglobulin G; vac = vaccination; CRP = C-reactive protein.

## Data Availability

The data that support the findings of this study are available from the corresponding author upon reasonable request.
